# Diagnostic value of fibrinogen to prealbumin ratio and gamma-glutamyl transpeptidase to platelet ratio in the progression of AFP-negative hepatocellular carcinoma

**DOI:** 10.1186/s12935-020-1161-y

**Published:** 2020-03-12

**Authors:** Li Huang, Zhuning Mo, Zuojian Hu, Linyan Zhang, Shanzi Qin, Xue Qin, Shan Li

**Affiliations:** 1 Department of Clinical Laboratory, First Affiliated Hospital of Guangxi Medical University, Nanning, 530021 Guangxi Zhuang Autonomous Region China; 2 Department of Blood Transfusion, The People’s Hospital of Guangxi Zhuang Autonomous Region, Nanning, Guangxi Zhuang Autonomous Region China

**Keywords:** Fibrinogen to prealbumin ratio, Gamma-glutamyl transpeptidase to platelet ratio, Diagnostic, AFP-negative hepatocellular carcinoma

## Abstract

**Background:**

This study aimed to comprehensively assess the diagnostic value of fibrinogen to prealbumin ratio (FPR) and gamma-glutamyl transpeptidase to platelet ratio (GPR) as single markers or in combination in patients with alpha-fetoprotein-negative (AFP-negative) hepatocellular carcinoma (HCC).

**Methods:**

A total of 199 healthy controls and 515 AFP-negative patients were enrolled in this study, including 180 HCC inpatients, 151 liver cirrhosis (LC) patients, and 184 chronic hepatitis (CH) cases. Mann–Whitney U or Kruskal–Wallis H test were used to analyze differences between groups in laboratory parameters and clinicopathological features. The diagnostic value of FPR and GPR, alone or in combination, in AFP-negative HCC (AFP-NHCC) patients was determined via a receiver operating characteristic (ROC) curve.

**Results:**

The levels of FPR and GPR were gradually increased in the development of AFP-NHCC and positively correlated with the tumor size and Barcelona Clinic Liver Cancer (BCLC) stages. Moreover, GPR was associated with Edmondson–Steiner grades. After univariate logistic regression analysis, FPR and GPR remained independent predictors of adverse outcomes. The combination of FPR and GPR had a good ability to detect AFP-NHCC from the control group (area under curve [AUC] = 0.977), AFP-negative CH (AUC = 0.745), and AFP-negative LC (AUC = 0.666). FPR combined with GPR possessed a larger area (0.943, 0.971) and sensitivity (87.50%, 89.81%) than FPR or GPR alone for differentiating AFP-NHCC with tumor size < 3 cm or at the BCLC-A stage.

**Conclusions:**

The pretreatment levels of FPR and GPR played vital roles in the development of AFP-NHCC, especially in patients with early or small AFP-NHCC.

## Background

Hepatocellular carcinoma (HCC) is the sixth-most commonly diagnosed cancer and the fourth leading cause of tumor-related death worldwide in 2018 [1]. Accumulated evidence demonstrates that inefficient diagnosis of HCC is still a major cause of high mortality, especially in patients harboring early or small HCC [2]. Surveillance guidelines for patients with a high risk of developing HCC primarily relied on ultrasound imaging and alpha-fetoprotein (AFP) [3]. Currently, serum AFP remains the most important and commonly serological diagnostic biomarker, but about 30–40% of overall HCC patients have normal AFP levels (< 20 ng/mL) [4]. This is referred to as AFP-negative hepatocellular carcinoma (AFP-NHCC) [5]. Even though the proportion of AFP-negative was as high as 15–30% in advanced patients, the American Association for the Study of Liver Diseases updated their practice guidelines in 2011, saying that AFP was no longer recommended for the detection of early HCC [6]. Although imaging technology has greatly improved the level of HCC detection, ultrasound images often fail to recognize small HCC nodules or distinguish malignant nodules from benign ones [7, 8], and the diagnosis rate for patients with AFP-NHCC is only 10.4% [9]. Patients with AFP-NHCC often have mild clinical symptoms, so other tumor markers are indispensable for its diagnosis, especially in the early stage of the disease. An array of numerous alternative yet costly tumor markers have been proposed to screen liver cancer, including Golgi protein 73 (GP73) [10], glypican-3 (GPC-3) [11], protein induced by vitamin K absence or antagonist-II (PIVKAII) [12], and some MicroRNAs [13]. However, none have shown sufficient sensitivity and/or specificity to meet the clinical routine practice requirements for the early diagnosis HCC [14]. Therefore, novel biomarkers with more economical, accurate, and useful predictions for the early diagnosis of HCC are urgently needed, especially for AFP-negative patients.

An increasing amount of research has pinpointed systematic inflammation and abnormal metabolism involved in the diagnosis and progression of HCC, such as fibrinogen (Fib) [15], prealbumin (PA) [16], platelet (PLT) [17], gamma-glutamyl transferase (GGT) [18], and combinations of several single markers in the forms of ratios [19, 20]. Indeed, Fib promotes the synthesis of proinflammatory cytokines and fibroblast growth factors to induce the malignant proliferation of tumor cells and accumulates vascular endothelium to enhance tumor angiogenesis [21, 22]. Zhang et al. [23] reported that hyperfibrinogenemia is relevant to advanced tumor stages and poor survival in patients with HCC. PA has a short half-life as a predictor of inflammatory stress and nutritional status [24], making it an effective biomarker for morbidity, mortality and tumor progression [25]. Therefore, fibrinogen to prealbumin ratio (FPR), which combines Fib and PA, has been used to predict clinical efficacy and outcome for several types of human cancers [20, 26]. In addition, the preoperative FPR can independently predict recurrence-free survival and overall survival and help identify HCC patients who could benefit from adjuvant chemotherapy [27]. Therefore, FPR may be a potent prognostic indicator for AFP-NHCC patients.

Gamma-glutamyl transpeptidase (GGT) to platelet ratio (GPR) was proposed by Lemoine et al. as a novel predictor for liver disease [28]. Ample evidence suggested that GPR was a good predictor for the diagnosis and prognosis of hepatitis liver, hepatic fibrosis, and hepatocellular carcinoma. Wang et al. [29] pointed out that the sensitivity and specificity of GPR in diagnosing liver inflammation was as high as 83.47% and 61.33%, respectively. The GPR showed better diagnostic accuracy than the aspartate transaminase to platelet ratio index (APRI) and the fibrosis index based on four factors (FIB-4) in assessing liver fibrosis in chronic hepatitis B (CHB) patients in West African populations [28]. Ke et al. [30] reported that GPR could predict complications in HCC patients undergoing minor liver resection. Hence, we hypothesized that this biomarker might have a better diagnostic value in the progression of AFP-NHCC.

To date, several studies have researched the value of FPR and GPR in the prognosis of HCC. However, there is a lack of data about the diagnostic value of FPR and GPR for the development of AFP-NHCC patients. Thus, this study evaluates whether the FPR and GPR could be used as predictive markers for patients with AFP-NHCC.

## Material and method

### Patients

180 HCC patients, 184 CH patients, and 151 LC patients who were all AFP-negative (< 20 ng/mL) were continuously recruited from the First Affiliated Hospital of Guangxi Medical University between Jan 2012 and Oct 2019. The inclusion criteria of AFP-negative patients were as follows: (1) newly diagnosed with HCC, and verified by surgical histopathology examination; LC was diagnosed via pathological examination and typical morphology upon ultrasonography or computed tomography (CT) imaging; or CH patients were confirmed to have been infected with hepatitis B virus (HBV)/hepatitis C virus (HCV) for at least 6 months; (2) no blood-system diseases or immunity-related diseases; (3) no presence of other types of cancers; (4) no infectious diseases other than hepatitis B or C; and (5) no organic disease outside of the liver. We also enrolled 199 healthy individuals with no history of cancer and no clinical evidence of liver disease as a control group. This research was approved by the Ethics Committee of the First Affiliated Hospital of Guangxi Medical University, and informed consent was obtained from all the participants.

A total of 180 patients (range 28–78 years) with AFP-NHCC were recruited in this study, including 151 patients with positive hepatitis B surface antigen, 21 patients with hepatitis C virus positive liver diseases, and 8 subjects with other types of liver cancer. And serum HBV-DNA levels ≥ 500 IU/mL was achieved in 69 of 151 AFP-NHCC patients who were hepatitis B surface antigen positive. According to the Barcelona Clinic Liver Cancer (BCLC) staging system, 108 patients (60.0%) had stage A, 53 (29.4%) had stage B, and 19 (10.6%) had stage C. Of the 151 cirrhosis patients, 130 were positive for hepatitis B surface antigen, 10 were positive for hepatitis C virus antibody, and 11 were other causes of cirrhosis, respectively. Subjects with hepatitis B surface antigen-positive were 159 in AFP-negative CH group, the rest were hepatitis C virus antibody-positive patients (25/184).

### Data detection and acquisition

All of the corresponding data for this study was extracted from the hospital’s electronic medical records before each qualified participant’s operation, including sex, age, white blood cells (WBC), platelets (PLT), hemoglobin (Hb), fibrinogen (Fib), prealbumin (PA), alpha-fetoprotein (AFP), alkaline phosphatase (ALP), total bilirubin (TBIL), alanine aminotransferase (ALT), aspartate amino transferase (AST), and gamma-glutamyl transpeptidase (GGT). Plasma fibrinogen concentrations were determined by the Clauss method using a Sysmex CA7000 automatic coagulation analyzer. Blood tests were performed with a Beckman-Coulter LH 780 hematology analyzer (Beckman Coulter, Brea, CA). The levels of PA, AST, ALT, TBIL, ALP, and GGT were tested by a Hitachi 7600 automatic biochemical analyzer (Tokyo, Japan) produced by Shanghai Zhicheng Bio-Technology Co., Ltd. Hepatitis B surface antigens and hepatitis C antibodies were analyzed using a chemiluminescent immunoassay (Abbott GmBH Diagnostika, Wiesbaden-Delkenheim, Germany). The values of FPR and GPR were calculated using the following formulas: FPR = fibrinogen level/prealbumin level; GPR = gamma-glutamyl transpeptidase level/platelet count.

### Statistical analysis

None of the data met the criteria for a normal distribution based on a Kolmogorov–Smirnov test. The median and interquartile ranges were applied for non-normally distributed data. The Mann–Whitney U test or Kruskal–Wallis H test were conducted to detect differences between groups in laboratory parameters and clinical characteristics. A receiver operating characteristic (ROC) curve and the area under the curve (AUC) were determined by MedCalc statistical software (version 18.1.1). The SPSS16.0 statistical software package was used for data processing and analysis, using a significant level of 0.05.

## Result

### General information about study population

**Fig. 1:**
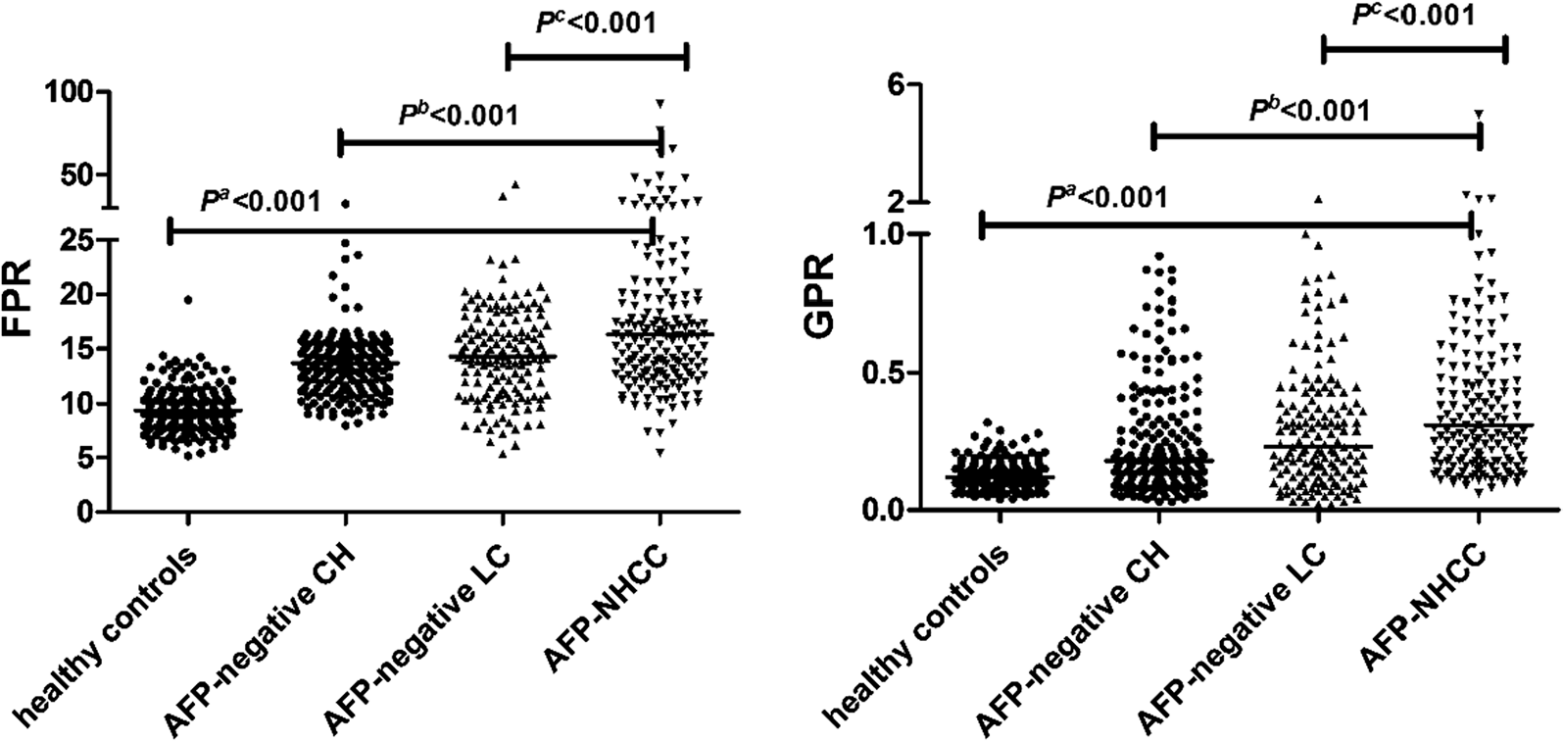
FPR and GPR among four groups. *FPR* fibrinogen to prealbumin ratio, *GPR* gamma-glutamyl transpeptidase to platelet ratio, *AFP-NHCC* alpha-fetoprotein-negative hepatocellular carcinoma, *AFP* alpha-fetoprotein, *CH* chronic hepatitis, *LC* liver cirrhosis. *p*^a^: AFP-NHCC group vs healthy controls; *p*^b^: AFP-NHCC group vs AFP-negative CH group; *p*^c^: AFP-NHCC group vs AFP-negative LC group

Basic information is shown in Table 1. The median PA of the 151 patients with AFP-negative LC was lower than those in other groups. Similarly, corresponding differences appeared in the levels of circulating PLT, WBC, Hb, and Fib. On the contrary, AFP- negative LC patients had higher TBIL and AST compared with other individuals. The value of FPR and GPR gradually increased with the development of AFP-NHCC (*p* < 0.001) (Fig. 1).

**Table 1:** Laboratory parameters in AFP-negative patients and healthy controls

Characteristics	Healthy controls (N = 199)		AFP-negative CH (N = 184)		AFP-negative LC (N = 151)		AFP-NHCC (N = 180)		*p*^a^	*p*^b^	*p*^c^
Gender (male/female)	169/30		145/39		115/36		163/17		0.097	0.002	< 0.001
Age (years)	50.00	42.00–56.00	39.50	32.00–48.75	46.00	37.00–54.00	50.00	43.25–58.00	0.212	< 0.001	< 0.001
WBC (× 10^9^/L)	6.10	5.38–6.87	6.21	5.24–7.19	5.76	3.98–7.39	6.00	5.06–7.47	0.837	0.793	0.027
Hb (g/L)	149.50	140.30–156.00	139.35	127.00–147.55	115.10	92.00–137.40	136.60	126.25–145.48	< 0.001	0.233	< 0.001
TBIL (μmol/L)	11.0	8.40–13.50	12.60	9.03–17.78	13.70	9.20–31.00	11.90	8.98–16.28	0.012	0.356	0.001
AST (U/L)	21.00	18.00–24.00	28.50	21.00–46.00	32.00	22.00–49.00	31.00	25.00–42.00	< 0.001	0.060	0.975
ALT (U/L)	20.00	16.00–25.00	32.00	20.25–77.75	27.00	18.00–45.00	33.00	25.00–46.75	< 0.001	0.667	0.007
PLT (× 10^9^/L)	241.00	207.40–280.30	203.00	169.27–246.38	166.70	96.80–244.20	169.75	129.12–214.42	< 0.001	< 0.001	0.547
Fib (g/L)	2.84	2.54–3.21	2.69	2.24–3.23	2.30	1.78–3.03	3.21	2.73–3.95	< 0.001	< 0.001	< 0.001
PA (mg/L)	305.10	280.00–338.30	204.45	166.92–244.12	165.30	111.50–218.80	195.00	165.18–235.55	< 0.001	0.031	< 0.001
GGT (U/L)	29.00	22.00–37.00	34.55	23.18–67.80	36.00	22.00–55.00	45.00	33.25–91.50	< 0.001	< 0.001	< 0.001
FPR	9.34	7.94–10.57	13.65	11.42–15.35	14.26	10.97–17.57	16.25	12.89–21.20	< 0.001	< 0.001	< 0.001
GPR	0.12	0.08–0.16	0.18	0.11–0.36	0.23	0.13–0.39	0.31	0.18–0.56	< 0.001	< 0.001	< 0.001

*WBC* white blood cells, *Hb* hemoglobin, *TBIL* total bilirubin, *ALT* alanine aminotransferase, *AST* aspartate amino transferase, *PLT* platelets, *Fib* fibrinogen, *PA* pre-albumin, *GGT* gamma-glutamyl transpeptidase, *FPR* fibrinogen to prealbumin ratio, *GPR* gamma-glutamyl transpeptidase to platelet ratio, *AFP-NHCC* alpha-fetoprotein-negative hepatocellular carcinoma, *AFP* alpha-fetoprotein, *CH* chronic hepatitis, *LC* liver cirrhosis

^a^AFP-NHCC group vs healthy controls (Mann–Whitney nonparametric U test)

^b^AFP-NHCC group vs AFP-negative CH group (Mann–Whitney nonparametric U test)

^c^AFP-NHCC group vs AFP-negative LC group (Mann–Whitney nonparametric U test)

### Correlation between FPR, GPR, and clinicopathological features in AFP-NHCC

As shown in Table 2, FPR and GPR were both associated with Barcelona Clinic Liver Cancer (BCLC) stages, but not related to the Child–Pugh grade, tumor encapsulation, or tumor multiplicity (all *p* > 0.05). Although 22 specimens were not clearly classified by Edmondson–Steiner grade, GPR showed a significant difference between the three subgroups of AFP-NHCC stages (*p *= 0.032).

**Table 2:** Correlation between clinicopathological features and FPR and GPR in AFP-NHCC

	Number (%)	FPR	*p*	GPR	*p*
Edmondson–Steiner grade					
I	19 (10.6)	13.56 (11.27–26.66)	0.345	0.47 (0.33–0.79)	0.032
II	114 (63.3)	15.99 (13.06–21.11)		0.300 (0.17–0.59)	
III	25 (13.9)	17.27 (14.07–21.84)		0.300 (0.19–0.50)	
Unknown	22 (12.2)	16.94 (13.59–20.38)		0.27 (0.14–0.39)	
BCLC stage					
A	108 (60.0)	15.65 (12.54–19.03)	0.004	0.29 (0.17–0.48)	0.024
B–C	72 (40.0)	17.14 (13.86–27.02)		0.37 (0.18–0.73)	
Child–Pugh grade					
A	162 (90.0)	16.12 (12.79–20.35)	0.334	0.30 (0.18–0.54)	0.137
B–C	18 (10.0)	17.00 (12.93–33.08)		0.42 (0.24–0.69)	
Tumor size (cm)					
< 3	40 (22.2)	14.22 (11.52–16.84)	0.001	0.22 (0.13–0.30)	< 0.001
≥ 3	140 (77.8)	16.99 (13.63–23.75)		0.37 (0.19–0.62)	
Tumor encapsulation					
Complete	123 (68.3)	16.30 (12.74–21.24)	0.721	0.31 (0.18–0.55)	0.384
None	57 (31.7)	15.37 (13.22–21.59)		0.34 (0.19–0.62)	
Tumor multiplicity					
Single	167 (92.8)	16.30 (12.80–21.27)	0.471	0.32 (0.18–0.57)	0.437
Multiple	13 (7.2)	14.07 (13.81–18.34)		0.27 (0.17–0.41)	

*FPR* fibrinogen to prealbumin ratio, *GPR* gamma-glutamyl transpeptidase to platelet ratio, *AFP-NHCC* alpha-fetoprotein-negative hepatocellular carcinoma, *BCLC* Barcelona Clinic Liver Cancer

### Logistic regression used to distinguish AFP-NHCC from controls

As shown in Table 3, the correlation between AFP-NHCC and some potential risk factors were analyzed using binary logistic regression, such as gender, age, TBIL, AST, ALT, ALP, FPR, and GPR. In the univariate analysis,“whether suffering from AFP-NHCC” was the response variable, these potential risk parameters were used as corresponding input variables one by one. In consideration of other confounding factors and the impact of a suppressor effect, variables with a significant value of *p* < 0.05 were subjected to a multivariate analysis and further screened by enter method to evaluate their independent effect. Odd ratio (OR) and 95% confidence interval (CI) were also calculated for each other. After the univariate analysis, several significant parameters were chosen as potential independent predictors for further multivariate analysis, including TBIL (OR = 1.086, 95% CI = 1.039–1.134, *p* < 0.001), ALT (OR = 1.133, 95% CI = 1.101–1.167, *p* < 0.001), AST (OR = 1.232, 95% CI = 1.175–1.293, *p* < 0.001), ALP (OR = 1.023, 95% CI = 1.013–1.033, *p* < 0.001), FPR (OR = 2.082, 95% CI = 1.783–2.430, *p* < 0.001), and GPR (OR = 1.748 × 10^8^, 95% CI = 2.003 × 10^6^–1.525 × 10^10^, *p* < 0.001). After adjusting for these six predictors, the results of the analysis demonstrated that FPR (*β* = 0.841, *p* < 0.001), GPR (*β* = 15.927, *p* < 0.001), and AST (*β* = 0.078, *p* = 0.023) were still important indicators closely related to the occurrence of AFP-NHCC. The optimal model for distinguishing AFP-NHCC patients from the control group was established through integration (logit *P* = 0.841 × FPR + 15.927 × GPR + 0.078 × ALT − 17.909). For this model, the AUC value was 0.981 (0.961 to 0.992), while the sensitivity and specificity were 92.22% and 97.99%, respectively.

**Table 3:** Univariate and multivariate analyses used for differentiating significant predictors to distinguish AFP-NHCC from healthy controls

Variables	Univariate analysis			Multivariate analysis		
	OR	95% CI	*P*-value	OR	95% CI	*p*-value
Gender	0.588	0.312–1.106	0.099			
Age(y)	1.013	0.992–1.035	0.232			
TBIL	1.086	1.039–1.134	< 0.001	1.110	0.985–1.250	0.086
AST	1.232	1.175–1.293	< 0.001	1.079	0.969–1.200	0.166
ALT	1.133	1.101–1.167	< 0.001	1.081	1.011–1.157	0.023
ALP	1.023	1.013–1.033	< 0.001	0.997	0.970–1.024	0.806
FPR	2.082	1.783–2.430	< 0.001	2.320	1.824–2.949	< 0.001
GPR	1.748 × 10^8^	2.003 × 10^6^–1.525 × 10^10^	< 0.001	8.260 × 10^6^	5.031 × 10^3^–1.356 × 10^10^	< 0.001

*TBIL* total bilirubin, *ALT* alanine aminotransferase, *AST* aspartate amino transferase, *ALP* alkaline phosphatase, *FPR* fibrinogen to pre-albumin ratio, *GPR* gamma-glutamyl transpeptidase to platelet ratio, *CI* confidence interval, *OR* odd ratio, *AFP-NHCC* alpha-fetoprotein-negative hepatocellular carcinoma

### Evaluating the diagnostic value of FPR, GPR between AFP-NHCC and other subjects

**Fig. 2:**
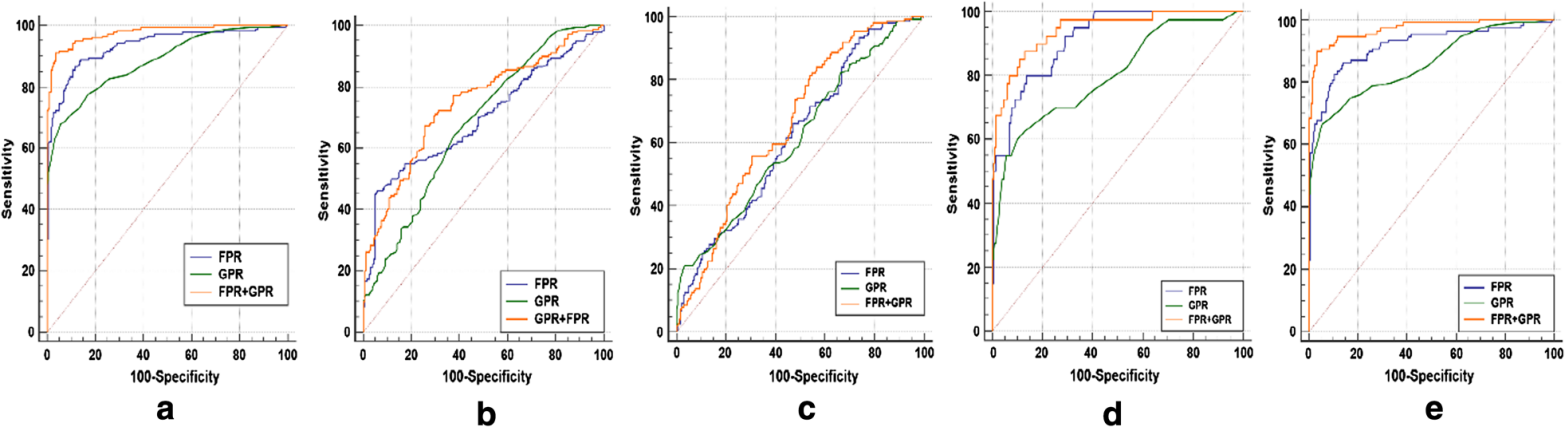
The diagnostic value of FPR and GPR for distinction between AFP-NHCC patients from other groups. **a** AFP-NHCC patients vs healthy controls. **b** AFP-NHCC patients vs AFP-negative CH patients. **c** AFP-NHCC patients vs AFP-negative LC patients. **d** AFP-NHCC patients with tumor size ≤ 3 cm vs healthy controls. **e** AFP-NHCC patients with BCLC-A stage vs healthy controls. *FPR* fibrinogen to prealbumin ratio, *GPR* gamma-glutamyl transpeptidase to platelet ratio, *AFP-NHCC* alpha-fetoprotein-negative hepatocellular carcinoma, *AFP* alpha-fetoprotein, *CH* chronic hepatitis, *LC* liver cirrhosis, *BCLC* Barcelona Clinic Liver Cancer

The results of the ROC curve analysis are shown in Table 4 and Fig. 2. The AUC value of FPR (AUC = 0.935) and GPR (AUC = 0.884) had a good diagnostic ability for distinguishing AFP-NHCC patients from controls. The sensitivity and specificity of the combination of FPR and GPR was increased to 91.11% and 96.48%, respectively. Compared to healthy controls, the AUC value of combination for FPR and GPR was 0.977 (95% CI = 0.957–0.990, positive likelihood ratio [PLR] = 25.90, negative likelihood ratio [NLR] = 0.09), positive predictive value [PPV] = 95.9%, negative predictive value [NPV] = 92.3%). Meanwhile, the AUC value of the combination of FPR and GPR in AFP-NHCC patients with tumor size < 3 cm was 0.943 (95% CI = 0.906–0.969), while the sensitivity and specificity was 87.50% and 86.93%, respectively. In the BCLC-A stage, the values of AUC, sensitivity, and specificity were 0.971, 89.81%, and 96.48%, respectively.

**Table 4:** Diagnostic efficacy of FPR and GPR used alone or in combination in differentiating patients with AFP-NHCC from other patients

	Cutoff	Sensitivity (%)	Specificity (%)	PLR	NLR	PPV (%)	NPV (%)	AUC (95% CI)	*p*
FPR^a^	11.57	86.67	88.44	7.50	0.15	87.2	88.0	0.935 (0.905–0.957)	< 0.001
GPR^a^	0.21	67.78	94.47	12.26	0.34	91.7	76.4	0.884 (0.848–0.915)	< 0.001
FPR^a^ + GPR^a^	0.44	91.11	96.48	25.90	0.09	95.9	92.3	0.977 (0.957–0.990)	< 0.001
FPR^b^	16.70	46.11	94.02	7.71	0.57	88.3	64.1	0.696 (0.646–0.743)	< 0.001
GPR^b^	0.23	64.44	61.96	1.69	0.57	62.4	64.0	0.677 (0.626–0.725)	< 0.001
FPR^b^ + GPR^b^	0.55	67.22	74.46	2.63	0.44	72.0	69.9	0.745 (0.697–0.789)	< 0.001
FPR^c^	20.72	26.11	93.38	3.94	0.79	82.5	51.5	0.623 (0.568–0.675)	< 0.001
GPR^c^	0.20	68.89	43.71	1.22	0.71	59.3	54.1	0.617 (0.563–0.670)	< 0.001
FPR^c^ + GPR^c^	0.42	46.11	81.46	2.49	0.66	74.8	55.9	0.666 (0.612–0.716)	< 0.001
FPR^d^	11.30	80.00	86.43	5.90	0.23	54.2	95.6	0.914 (0.871–0.946)	< 0.001
GPR^d^	0.19	60.00	89.95	5.97	0.44	54.5	91.8	0.801 (0.745–0.850)	< 0.001
FPR^d^ + GPR^d^	0.15	87.50	86.93	6.70	0.14	57.4	97.2	0.943 (0.906–0.969)	< 0.001
FPR^e^	11.26	86.11	85.93	6.12	0.16	76.9	91.9	0.916 (0.879–0.944)	< 0.001
GPR^e^	0.21	66.67	94.47	12.06	0.35	86.7	83.9	0.859 (0.814–0.896)	< 0.001
FPR^e^ + GPR^e^	0.16	89.81	96.48	25.53	0.11	93.3	94.6	0.971 (0.945–0.987)	< 0.001

*FPR* fibrinogen to prealbumin ratio, *GPR* gamma-glutamyl transpeptidase to platelet ratio, *PLR* positive likelihood ratio, *NLR* negative likelihood ratio, *PPV* positive predictive value, *NPV* negative predictive value, *CI* confidence interval, *AUC* area under curve, *AFP-NHCC* alpha-fetoprotein-negative hepatocellular carcinoma, *AFP* alpha-fetoprotein, *CH* chronic hepatitis, *LC* liver cirrhosis, *BCLC* Barcelona Clinic Liver Cancer

^a^AFP-NHCC patients vs healthy controls

^b^AFP-NHCC patients vs AFP-negative CH patients

^c^AFP-NHCC patients vs AFP-negative LC patients

^d^AFP-NHCC patients with tumor size < 3 cm vs healthy controls

^e^AFP-NHCC patients with BCLC-A stage vs healthy controls

To predict AFP-NHCC, the optimal cut-offs of FPR were 16.70 and 20.72, for the diagnosis of AFP-negative CH (AUC = 0.696, sensitivity = 46.11%, specificity = 94.02%, PPV = 88.3%, and NPV = 64.1%), and AFP-negative LC (AUC = 0.623, sensitivity = 26.11%, specificity = 93.38%, PPV = 82.5%, and NPV = 51.5%), respectively. The optimal cut-offs of GPR were 0.23 and 0.20, for the diagnosis of AFP-negative CH (AUC = 0.677, sensitivity = 64.44%, specificity = 61.96%, PPV = 62.4%, and NPV = 64.0%), and AFP-negative LC (AUC = 0.617, sensitivity = 68.89%, specificity = 43.71%, PPV = 59.3%, and NPV = 54.1%), respectively. For the diagnosis of AFP-NHCC, the combined use of FPR and GPR resulted in a greater AUC (0.745 for AFP-negative CH and 0.666 for AFP-negative LC) than FPR or GPR alone.

## Discussion

Early diagnosis of HCC is closely associated with its prognosis, which can substantially enhance the 5-year survival rate of patients [31]. AFP, as a regulatory surveillance indicator of HCC, is limited in detecting HCC by poor diagnostic efficiency [32]. Thus, novel tumor biomarkers should be made to help the clinical diagnosis of AFP-NHCC. Numerous inflammatory response markers have been presented as effective, economical, and reliable indicators for the diagnosis and prognosis of AFP-NHCC. These markers include PA [33], D-Dimer [33], C-reactive protein [34], platelet-lymphocyte ratio [34], lactate dehydrogenase [35] and GGT [35]. However, the predictive roles of FPR and GPR in AFP-NHCC remained unknown. Therefore, this study investigated FPR and GPR to assess whether these parameters related to the progression of AFP-NHCC and could have diagnostic value.

Research has proven that coagulation and nutritional status may affect the progression of HCC patients. Fibrinogen, as an important coagulation factor, synthesized by hepatocytes. Zhu et al. [36] demonstrated that the mRNA levels of fibrinogen were elevated both in cell lines and tissues, and increased plasma fibrinogen levels were associated with tumor thrombosis. On the other hand, gathering evidences have suggested that prealbumin is decreased and closely related with various malignancies, including liver cancer [37]. Therefore, the levels of FPR may be up-regulated in cancer patients. Indeed, preoperative FPR levels in the AFP-NHCC group were significantly higher than those in other groups, which concurred with previous reports. Sun et al. [20] reported that circulating FPR was significantly higher in patients harboring colorectal cancer than in benign and healthy subjects. Wang et al. [38] displayed that the levels of the prealbumin to fibrinogen ratio were reduced in severe acute pancreatitis and inversely proportional to the progression of acute pancreatitis. Another study by Zhang found that patients with low FPRs were observed to have a long survival, and the prognosis of stage III FPR-low gastric cancer patients undergoing chemotherapy was significantly superior to the patients without chemotherapy treatment [26]. FPR was also a prognostic marker in HCC, with a high FPR related to decreased survival and longer overall survival [27]. Moreover, several studies revealed that preoperative FPR was significantly correlated with clinical parameters in various solid tumors [20, 26]. Two scholars, Hu [39] and Zhang [27], found that FPR levels were higher in patients with large tumor sizes and advanced stages of HCC. The present study also demonstrated that FPR moderately increased with tumor size and BCLC stage of AFP-NHCC, demonstrating that this biological indicator may be related to the invasive phenotype of the disease.

As a crucial enzyme in glutathione metabolism, Gamma-glutamyl transferase (GGT) was continually elevated in metabolic-induced hepatic injury [40], was observed in this study. Salvatore et al. [41] demonstrated that the level of serum GGT elevated with the process of liver carcinogenesis and promoted tumor progression in an HCC animal model of male Wistar rats. Serum levels of GGT could also help with the selection of further treatment and clinical outcomes for patients with HCC [42]. Carr et al. [43] found that patients with significantly high GGT values were prone to poor overall survival in cases of low AFP HCC. Thrombocytopenia frequently occurs in chronic liver disease, mainly because of accelerated platelet destruction caused by hypersplenism and hepatocyte damage leading to a decrease in thrombopoietin [17]. These two opposite variables, GGT and PLT, were applied to GPR to further amplify and improve its predictive value. Park et al. [44] found that the relative risk of HCC development in the low-GPR group was lower than that of the high-GPR group. Compared to low-GPR patients, subjects with a higher GPR possessed a higher probability of cirrhosis, being a worse outcome [45]. The above studies indicate that GPR level increased with the severity of liver damage, which matches the findings of the present study as well. Our found that GPR levels were raised in the development of AFP-NHCC. In addition, regarding the relationship between GPR and clinical characteristics, it has been proven that patients with an elevated GPR had a higher probability of larger tumor size than individuals with a lower GPR [45]. Another study by Hu et al. [39] displayed that the levels of GPR were positively correlated with BCLC stages. Similar to previous studies, our results indicated that GPR levels increased with the progression of tumor size and cancer stage, including both the Edmondson–Steiner grade and BCLC stage.

The present study first assessed the preoperatory FPR and GPR levels to evaluate their diagnostic efficacy in the development of AFP-NHCC. Both FRP and GPR significantly increased with the progression of AFP-NHCC as well as tumor size and cancer stage. Moreover, high FPR and GPR levels were also independent poor outcome predictors in multivariate logistic regression analyses adjusted for potential predictors. These suggested that FPR and GPR might be important markers in the progression of patients with AFP-NHCC. FPR and GPR had good AUCs and sensitivity in identifying AFP-NHCC patients from controls. Many papers indicated that a series of biomarkers could help diagnose AFP-NHCC, showing the AUC of GP73 [46], AFP-L3 [46], and PIVKAII [47] used to distinguish AFP-NHCC from controls was 0.7811, 0.6094, and 0.856, respectively, while the sensitivity was 66%, 50%, and 76.3%, respectively. These AUC values were lower than FPR and GPR, and the sensitivity of FPR was superior to those biomarkers for detecting AFP-NHCC. In addition, the combination of FPR and GPR had a larger AUC than FPR or GPR alone for discriminating AFP-NHCC patients and healthy controls. One retrospective study by Best and colleagues revealed that the detection rate of a combination of PIVKAII and AFP-L3 was only 68.4% for patients with AFP-NHCC, which is lower than FPR and GPR combined. Zhang et al. [46] reported that the sensitivity achieved when using a combination of the assay results of AFP-L3 and GP73 were merely 40%, which was not as sensitive as the combination with FPR and GPR. Compared to the CHB group, the AUC and sensitivity of PIVKAII in AFP-NHCC patients was 0.73 and 51.02%, respectively, which was slightly lower than our results (0.745, 67.22%). In addition, FPR and GPR were also moderate predictors for distinguishing AFP-NHCC patients from AFP-negative LC or AFP- negative CH. Hence, the current results indicate that the combined use of FPR and GPR may improve the clinical diagnostic efficiency of differentiating AFP-NHCC from other groups.

Several limitations should be concerned. First, we failed to distinguish viral physical status and viral load from the stratified analyses due to the relatively small sample size. Second, AFP-negative cases were limited and all came from the same hospital, which may create bias in evaluating the predictive value of these markers. Third, personal information, including dietary habits and family histories, was not obtained, which may influence the final results. Hence, future prospective studies require multiple centers, a larger scale, and more detailed information to validate these results.

## Conclusion

The present study demonstrated that FPR and GPR were correlated with AFP-NHCC as well as tumor size and BCLC stage. The combination of FPR and GPR, as economic, simple, effective, and promising biomarkers, possessed a high diagnostic efficiency in the progression of patients with AFP-NHCC, especially in patients with early or small AFP-NHCC.

## Data Availability

The datasets supporting the conclusions of this article is included within the article.

## Abbreviations

WBC: White blood cells
Hb: Hemoglobin
PLT: Platelets
TBIL: Total bilirubin
ALT: Alanine aminotransferase
AST: Aspartate amino transferase
Fib: Fibrinogen
PA: Prealbumin
ALP: Alkaline phosphatase
GGT: Gamma-glutamyl transpeptidase
ROC: Receiver-operating characteristic curve
AUC: Area under curve
OR: Odd ratio
CI: Confidence interval
FPR: Fibrinogen to prealbumin ratio
GPR: Gamma-glutamyl transpeptidase to platelet ratio
AFP: Alpha-fetoprotein
LC: Liver cirrhosis
CH: Chronic hepatitis
HCC: Hepatocellular carcinoma
